# Acetylated Deoxynivalenol Generates Differences of Gene Expression that Discriminate Trichothecene Toxicity

**DOI:** 10.3390/toxins8020042

**Published:** 2016-02-06

**Authors:** Tadahiro Suzuki, Yumiko Iwahashi

**Affiliations:** Applied Microbiology Division, National Food Research Institute, National Agriculture and Food Research Organization (NARO), 2-1-12 Kannon-dai, Tsukuba, Ibaraki 305-8642, Japan; suzut@affrc.go.jp

**Keywords:** DON, acetylated derivative, toxicity, gene expression

## Abstract

Deoxynivalenol (DON), which is a toxic secondary metabolite generated by *Fusarium* species, is synthesized through two separate acetylation pathways. Both acetylation derivatives, 3-acetyl-DON (3ADON) and 15-acetyl-DON (15ADON), also contaminate grain and corn widely. These derivatives are deacetylated via a variety of processes after ingestion, so it has been suggested that they have the same toxicity as DON. However, in the intestinal entry region such as the duodenum, the derivatives might come into contact with intestinal epithelium cells because metabolism by microflora or import into the body has not progressed. Therefore, the differences of toxicity between DON and these derivatives need to be investigated. Here, we observed gene expression changes in the yeast *pdr5Δ* mutant strain under concentration-dependent mycotoxin exposure conditions. 15ADON exposure induced significant gene expression changes and DON exposure generally had a similar but smaller effect. However, the glucose transporter genes *HXT2* and *HXT4* showed converse trends. 3ADON also induced a different expression trend in these genes than DON and 15ADON. These differences in gene expression suggest that DON and its derivatives have different effects on cells.

## 1. Introduction

**Figure 1:**
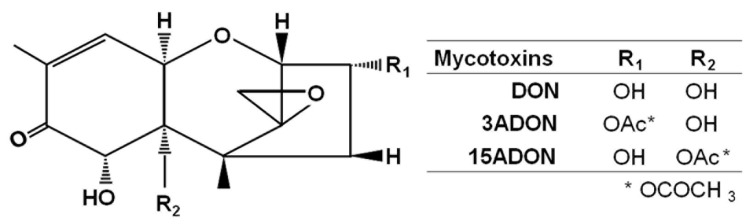
Deoxynivalenol and its derivatives.

**Figure 2:**
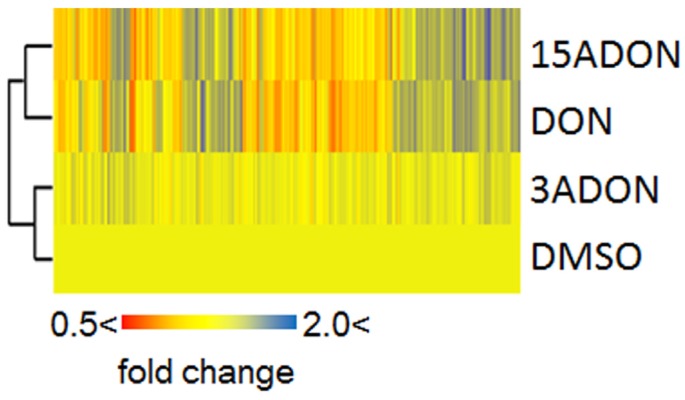
Comprehensive gene expression changes caused by mycotoxin exposure. DNA microarray data for the control sample (dimethyl sulfoxide; DMSO) and mycotoxin-exposed samples (25 mg/L (=25 ppm) deoxynivalenol; DON, 3-acetyl-DON; 3ADON, and 15ADON) were taken from a previous analysis (accession number GSE36954) [3]. These data were used to reconstruct a hierarchical map. Non-significant gene expression changes are indicated in yellow. Induced genes are represented in red; conversely, reduced genes are represented in blue.

Deoxynivalenol (DON) is a secondary metabolite synthesized by *Fusarium* species, and is a mycotoxin that has various toxic effects. DON, which is a type B trichothecene mycotoxin, is restricted in a number of countries, and regulatory limits have been established. The major activity of DON is binding to ribosome subunits, which affects cellular translation control. Because of this abnormal translation, cell proliferation is inhibited and disorder of physiological functions is induced. In the mammalian body, the region around the intestinal epithelia is very likely to be affected by DON. Cell lesions present as a toxicity phenotype through inflammation derived from enhanced inflammatory cytokines [1,2], and it is thought that DON induces abdominal pain or diarrhea. In yeast cells, which are used as a model in eukaryotic cell studies, some type B trichothecenes including DON affect the expression of ribosomal protein coding genes localized in both the cytosol and mitochondria [3]. Trichothecin, which belongs to the trichothecene mycotoxin family, has also been related to yeast mitochondrial gene expression changes [4]. However, DON exposure induces chlorosis in the leaves of *Arabidopsis thaliana* [5]. In this manner, DON shows various effects on different organisms. The DON synthesis pathway generates a number of derivatives that have this toxicity, though they differ in intensity. Acetylated DONs, such as 3-acetyl-DON (3ADON) and 15-acetyl-DON (15ADON) (Figure 1), are one type of derivative, and their environmental abundance ratios should not be ignored. Acetylated DONs are usually found at quite low levels, though high amounts of 3ADON compared with DON have occasionally been found in field experiments [6]. These acetylation types, 3A and 15A, are selectively decided in the DON synthesis pathway. Chemotypes, which are an expedient taxonomic designation, have been advocated for these acetylated products, and worldwide long-term experiments suggest that the composition of chemotypes changes repeatedly [7]. Therefore, it is necessary to better understand the characteristics of both DON and acetyl-DONs, and to consider their effects on organisms. In fact, one viewpoint is that the acetylation of DON is a transient detoxification mechanism for preventing autotoxicity. Indeed, 3-acetylation represents an effective detoxification, and previous experiments have indicated that 3ADON does not have any significant toxic phenotypes; however, 15ADON showed high toxicities to various cell lines [8,9,10]. Hence, except in fungal cells, it is thought that 15-acetylation enhances DON toxicity. In a previous study, we tested DON, 3ADON, 15ADON, nivalenol (NIV) and 4-acetyl-nivalenonl (4ANIV) using yeast cells, and investigated gene expression changes by DNA microarray (Figure 2) [3]. DON, 15ADON, and 4ANIV showed high toxicity, so acetylation was not linked to the degree of toxicity. Additionally, NIV, which shows high toxicity to several mammalian cell lines, did not present significant toxicity. *Chlamydomonas reinhardtii*, which is a green alga and has moderate sensitivity to NIV, was also used for a trichothecene exposure experiment [11]. However, 3ADON had no significant negative effects on any cell line.

Acetylated DON is imported from the digestive apparatus, and is then de-acetylated before it arrives at organs such as the liver. In such instances, it is thought that DON and its derivatives have a consistent toxicity mechanism against organisms. This suggests that a regulatory limit dependent on only the DON concentration is a problem because acetylated derivatives are ignored. Additionally, DON is de-epoxidized by intestinal microflora, and there is no convincing evidence that de-epoxy DON has toxic characteristics [12,13]. Therefore, the toxicity of these trichothecenes to an organism is considerably influenced by the organism’s microflora. Because digestion by microflora and import to the organs have not progressed much, the region around the duodenal intestinal epithelium is thought to be susceptible to inflammation. At the same time, this means that DON derivatives retain their structure, which implies that derivatives induce toxicity through their own structures. If this is the case, it is necessary to identify specific derivative-induced toxic characteristics. In our previous research, we compared the comprehensive gene expression changes in yeast cells exposed to DON or its derivatives by DNA microarray analysis [3]. Yeasts do not have a deacetylation enzyme that reacts to DON derivatives; thus, the gene expression changes should represent derivative-specific toxic characteristics if the efficiency of intermembrane transfer is ignored. In that research, a mutant strain of pleiotropic drug resistance (PDR) transporter 5, which contributes substantially to type B trichothecene export, was used, and sodium dodecyl sulfate was added to increase the membrane permeability of mycotoxins. Even so, 3ADON exposure did not present a significant toxic phenotype or induce gene expression changes. Thus, it seems likely that structural changes derived from acetylation generate toxicity differences. The European Food Safety Authority (EFSA) reported that 15ADON represented up to 15% of DON contamination of agricultural products [14]. Additionally, 15ADON has shown high toxicity in various cell studies [3,5,11,12], so it should not be ignored. However, it is uncertain whether the differences in gene expression correspond to toxicity level changes because a number of genes, such as regulatory genes that encode transcription factors, often show temporary or rapid changes. A time-series experiment could solve this problem, but highly toxic agents prevent cell growth over time. Therefore, the gene expression changes will not provide clear information. Another way to obtain continuous gene expression data is with a concentration-dependent exposure experiment, which, through gene expression changes, could provide new insight into the differences in toxicity characteristics between DON and its derivatives. Here, we conducted a concentration-dependent exposure experiment using gene data that were extracted from a previous DNA microarray analysis [3]. DNA microarray analysis revealed significant expression changes derived from high-toxicity materials such as DON or 15ADON. However, it was insufficient to discriminate the differences in toxic characteristics, although the changes were useful for obtaining toxicity information about trichothecene mycotoxins. Therefore, we examined several new genes that showed different expression changes between trichothecenes, and identified concentration-dependent gene expression trends that could distinguish each mycotoxin.

## 2. Results and Discussion

### 2.1. Influences of Pleiotropic Drug Resistance Transporter Regulation

**Figure 3:**
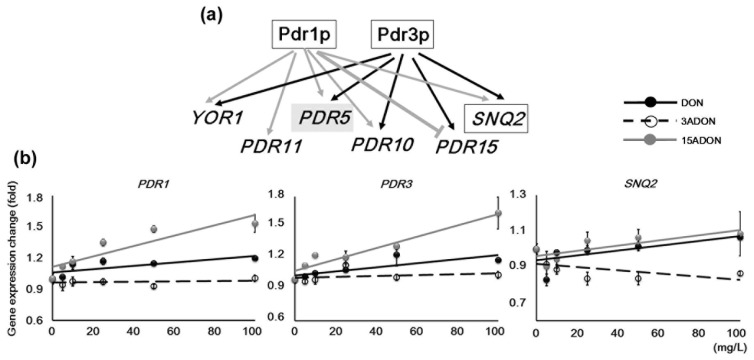
Expression changes of pleiotropic drug resistance (PDR) genes. (**a**) A simplified pleiotropic drug resistance regulation system, which was compiled by referring to a previous report [21]. Genes in boxes were examined in this study. Three capital letters with *Italics* indicates a gene; two small letters following a capital letter indicates a coding protein; (**b**) Concentration-dependent expression changes of PDR genes obtained by semi-quantitative polymerase chain reaction (PCR). *ACT1* expression data in each sample were used for normalization. Approximate trend lines are shown. Bars = standard error; *n* = 3. *p*-value of ANOVA < 0.01, except for *SNQ2*.

A pleiotropic drug resistance transporter gene-deletion mutant strain of yeast, *pdr5Δ*, was used in this study. Pdr5p is a plasma membrane ATP-binding cassette (ABC) transporter of yeast, and functions as an efflux pump of various chemicals [15,16,17,18]. The strain *pdr5* is a major DON-sensitive mutant, and a number of toxicity studies have been performed using this strain [3,18,19]. Additionally, it has been reported that the mutant of *SNQ2*, which is an ABC transporter (Table 1), has moderate sensitivity to mycotoxins [20]. Thus, both coding proteins, Pdr5p and Snq2p, are thought to be important for decreasing DON toxicity. The expression of these genes is regulated by the same transcription system as Pdr1p and Pdr3p (Figure 3a), so it is assumed that the expression of *SNQ2* is up-regulated by the DON family in the *pdr5Δ* mutant. However, in a previous DNA microarray analysis, the expression of these genes did not show many significant changes. It is unclear whether changes of mycotoxin concentration affect the function of the PDR system in yeast cells. This may result in misinterpretation of the toxicity of trichothecene mycotoxins using the *pdr5Δ* mutant. We therefore observed the *PDR1*, *PDR3*, and *SNQ2* expression trends under various mycotoxin conditions. The expression of these genes was induced under DON and 15ADON conditions (Figure 3b). *SNQ2* generally showed small expression changes (two-way factorial ANOVA; *p* = 0.30), though its expression was suppressed under 3ADON conditions (Student’s *t*-test with DON or 15ADON, *p* < 0.01) such that a precise view was not obtained. In contrast, relatively high induction of both *PDR1* and *PDR3* was observed under 15ADON conditions, while DON conditions showed lower induction compared with 15ADON. The expression of these transcription factors indicated that the toxicity of 15ADON was strong compared with that of DON. However, these expression change data did not reveal any hints to differentiate 15ADON from DON because the expression trends were similar. On the other hand, changing the mycotoxin concentrations did not lead to transient changes of gene expression patterns. Consequently, these data at least suggest that the yeast PDR system genes do not show transiently significant expression, which would prevent interpretation of the toxicity characteristics of these mycotoxins, and it is thought that experiments using *pdr5Δ* can reveal the toxicity of each mycotoxin adequately.

**Table 1:** Pleiotropic drug resistance-related genes observed in this study.

Systematic Name	Gene Symbol	Localization	Description
YGL013C	*PDR1*	cytoplasm, nucleus	Regulates pleiotropic drug resistance
YBL005W	*PDR3*	cytoplasm, nucleus	Transcriptional activator of the pleiotropic drug resistance network
YDR011W	*SNQ2*	plasma membrane	Plasma membrane ATP binding cassette (ABC) transporter

### 2.2. Stress-Responsive Redox Genes

**Figure 4:**
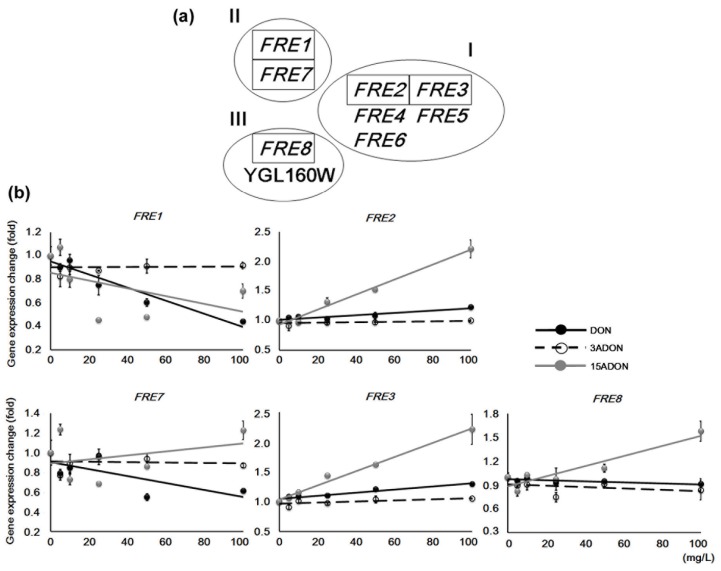
Expression changes of ferric reductase (*FRE*) genes. (**a**) Experimentally categorized families of *FRE* genes. Genes in boxes were examined in this study; (**b**) Concentration-dependent expression changes of *FRE* genes were identified by semi-quantitative PCR. *ACT1* expression data in each sample were used for normalization. Approximate trend lines are shown. Bars = standard error; *n* = 3. *p*-value of ANOVA < 0.01.

Since suitable *PDR* genes for discriminating DON and acetylated DON were not found, we focused on other stress response mechanisms. Yeast cells form an iron-sulfur (Fe-S) cluster that functions as an adjustor of redox reactions to prevent DNA damage caused by reactive oxygen species (ROS). To maintain this mechanism, cells import Fe^2+^, which is reduced from Fe^3+^, through cellular membrane transporters. Yeast has *FRE* genes that encode ferric/cupric reductases that localize to the plasma membrane, and these genes are roughly categorized into three families [22] (Table 2, Figure 4a). The enzymatic activities of both Fre1p and Fre2p cover almost all (>90%) iron ion reduction activity at the plasma membrane [23]. Mammalian cells such as intestinal epithelium cells have a similar function; enteral Fe^3+^ is reduced to Fe^2+^, which is preserved as ferritin or used to regulate intravital oxygenic products. However, Fre1p and Fre2p are similar to gp91, a large subunit of cytochrome b-245, which is a component of human NADPH oxidase [23,24]. This human component synthesizes superoxide through the addition of an electron at the membrane surface, and this superoxide kills harmful extracellular microorganisms. A lack of gp91 causes depression of bactericidal activity, and induces X-linked chronic granulomatous disease. Conversely, excess gp91 activity causes ROS stress to its own cells. Thus, the monitoring of gp91 can help us understand changes in cellular oxidative stress. If Fre1p and Fre2p have the same function, *FRE* genes have the potential to be markers for the stress response at the cellular membrane surface of yeast. Therefore, we observed the expression of *FRE1* and *FRE2* (Figure 4b). Three additional genes, *FRE7*, *FRE3*, and *FRE8*, were also tested under the same conditions. *FRE7*, which is a *FRE1* family member, did not show many significant changes, though DON exposure decreased its expression. For *FRE1*, more than 25 mg/L of both DON and 15ADON caused a significant reduction of expression. The expression of *FRE2* and *FRE3* (family I) showed significant concentration-dependent induction under 15ADON conditions, while DON exposure resulted in the same trend of a small expression increase. Although it had smaller expression changes, *FRE8*, which belongs to a different group from the above families, also showed a similar expression pattern to *FRE2* and *FRE3*. Taken together, both DON and 15ADON reduced *FRE1* expression, and induced *FRE2* and *FRE3*. Moreover, for *FRE2* and *FRE3*, 15ADON-induced gene expression changes were higher than those of DON. These results indicated contrasting expression between *FRE1* and *FRE2*, and suggested that their roles or functions are different. This study might be the first report of contrasting gene expression patterns for *FRE1* and *FRE2*, although it is known that they are induced by iron deficiency. Nonetheless, the *FRE1* and *FRE2* genes were not suitable for discriminating the toxicity characteristics of DON and 15ADON because the expression patterns of both were partially similar, while the expression trends of *FRE3* corresponded to those of *FRE2*. As for *FRE7*, the expression trends differed under DON and 15ADON exposure. However, it would be difficult to discriminate the influence of each because the 15ADON-derived gene expression changes were not stable. Additionally, a lack of *FRE7* has no specific effect [25], so Fre7p is thought to be an accessory enzyme. Overall, trichothecene exposure caused *FRE* gene expression changes, although this does not necessarily mean they can be used as markers for evaluating trichothecene toxicity. On the surface, the role of Fre seems contrary to that of gp91. However, both mechanisms contribute to stress reduction through redox reactions; thus, redox mechanisms are thought to be important for maintaining cellular components and processes across species.

**Table 2:** *FRE* family genes observed in this study.

Systematic Name	Gene Symbol	Group *	Induction with		Localization	Description
			Low Level Iron	Low Level Copper		
YLR214W	*FRE1*	II	+	++	plasma membrane	Ferric reductase and cupric reductase
YKL220C	*FRE2*	I	++	+		Ferric reductase
YOR381W	*FRE3*	I	++	+		Ferric reductase
YOL152W	*FRE7*	II	-	++		Ferric reductase and cupric reductase
YLR047C	*FRE8*	III	-	-		Similarity to ferric/cupric reductases

* Group numbers were established in experiments [22]. +, ++ and - indicates the gene induction levels. +: induction; ++: high level induction; -; not induced or not confirmed.

### 2.3. Genes Specifically Responsive to 15ADON

**Figure 5:**
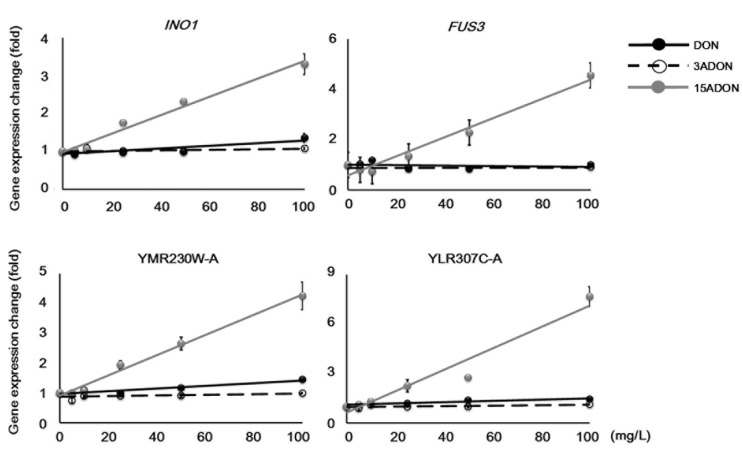
15ADON-specific gene expression changes. Concentration-dependent gene expression changes were identified by semi-quantitative PCR. *ACT1* expression data in each sample were used for normalization. Approximate trend lines are shown. Bars = standard error; *n* = 3. *p*-value of ANOVA < 0.01.

In investigating gene expression trends, we found various types of concentration-dependent changes. For example, the expression of several genes was reduced under higher concentrations but was induced under low concentration conditions. Such inconsistent expression changes can mislead gene expression analysis. To make interpretation easier, we next examined several genes that showed 15ADON-specific expression changes (Table 3, Figure 5).

**Table 3:** Uncategorized genes showing 15ADON-specific expression changes.

Systematic Name	Gene Symbol	Localization	Description
YBL016W	*FUS3*	cytoplasm, nucleus	Mitogen-activated serine/threonine protein kinase
YJL153C	*INO1*	cytoplasm	Inositol-3-phosphate synthase
YLR307C-A	-	unknown	Uncharacterized
YMR230W-A	-	unknown	Uncharacterized

*INO1* encodes an inositol-3-phosphate synthase involved in the synthesis of inositol phosphates (IPs), which are utilized in the sphingolipid metabolic pathway for maintaining various cellular processes. In the sphingolipid metabolic pathway, IPs are used as not only cellular components but also cell proliferation signals, and normal IP regulation is essential to maintain normal cellular processes. In a previous toxicity evaluation study of aflatoxin B_1_ (AFB_1_) using the yeast mutant strain of *PTC1*, which encodes an inhibitory regulation protein of the mitogen-activated protein kinase (MAPK) pathway, we observed significant up-regulation of *INO1* [26]. Furthermore, in that study, the down-regulation of sphingolipid metabolism pathway genes was examined. In addition, it has been reported that fumonisin B_1_ (FB_1_), which is a mycotoxin synthesized by *Fusarium* sp., also inhibits ceramide synthase located in the sphingolipid metabolism pathway [27]. Because 15ADON induced *INO1* in a concentration-dependent manner, it may affect the sphingolipid metabolism pathway such as AFB_1_ or FB_1_. *FUS3* also encodes a serine/threonine-specific kinase of the MAPK pathway, which is activated as a stress response. Additionally, activation of Fus3 induces G_1_ arrest of the cell cycle [28]. In this study, *FUS3* was induced by 15ADON exposure, suggesting cell cycle arrest. Given that DON-derived *FUS3* induction was quite low, *FUS3* might be a suitable marker for discriminating DON and 15ADON. The uncharacterized genes *YLR307C-A* and *YMR230W-A* showed similar results to *FUS3*. These genes do not have any known characteristics such as signal peptides, motifs, or transmembrane regions. However, they showed consistent expression trends, suggesting that they are influenced by some sort of functional gene damaged by mycotoxin exposure. Because *FUS3* and *YLR307C-A* showed small expression changes under DON conditions, these genes especially could be superior candidates as markers for 15ADON toxicity.

### 2.4. Glucose Transporter Genes Differentiate DON and its Derivatives

**Figure 6:**
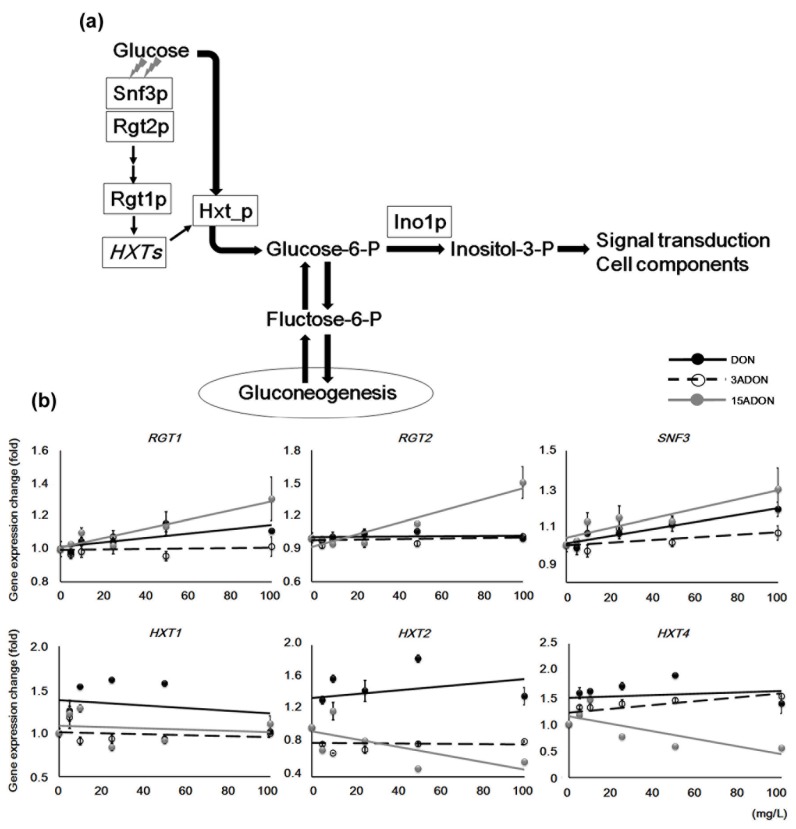
Expression changes in glucose import and utilization genes. (**a**) The transporter system and gluconeogenesis provide glucose, which is converted to inositol. Three capital letters with *Italics* indicate a gene; two small letters following a capital letter indicate a coding protein; (**b**) Concentration-dependent expression changes in glucose transporter and sensor or regulator genes were identified by semi-quantitative PCR. *ACT1* expression data in each sample were used for normalization. Approximate trend lines are shown. Bars = standard error; *n* = 3. *p*-values of ANOVA: *RGT1*, *p* < 0.03; other genes, *p* < 0.01.

In yeast cell studies, 15ADON causes a large number of gene expression changes compared with other type B trichothecenes, and the change levels are also significant [3]. DON induced relatively small expression changes compared with 15ADON despite showing similar expression trends, such that DON-specific expression changes were not easily identified. However, some DON-specific gene expression changes were observed. In this study, we focused on the hexose transporter genes, *HXT2* and *HXT4*, which localize to the plasma membrane such as *FRE* genes (Table 4, Figure 6a). The regulatory mechanisms of *HXT1*, *HXT2*, and *HXT4* have been well investigated, and they are generally induced by changes of glucose level [29,30,31]. Glucose is transported from the extracellular space, or synthesized by gluconeogenesis, and then converted to inositol. Because of this glucose usage circulation, *HXT* genes were examined (Figure 6b). *HXT1* and *HXT2* were induced by DON exposure, whereas the other mycotoxins did not show significant gene expression induction. In addition, *HXT2* and *HXT4* showed a repression trend under 15ADON exposure while the opposite trend was observed under DON exposure. In this way, the trends under DON and 15ADON were clearly different, although under 100 mg/L conditions, *HXT1* did not show any significant difference. Taken together, both *HXT2* and *HXT4* might be suitable genes for discriminating DON- and 15ADON-specific toxicity characteristics. Additionally, 3ADON exposure caused *HXT2* repression and *HXT4* induction, although the changes were small. These expression patterns differed from those of DON and 15ADON, so *HXT2* and *HXT4* could also be valuable genes for discriminating 3ADON-specific toxicity. The HXT genes have different regulation mechanisms; *HXT1* encodes a low affinity glucose transporter that is induced by >1% glucose conditions, while the rest are high affinity glucose transporters that are induced by <0.2% glucose condition [31]. Additionally, our results suggested novel characteristics for these genes.

**Table 4:** Glucose-sensing and importer genes observed in this study.

Systematic Name	Gene Symbol	Localization	Description
YKL038W	*RGT1*	nucleus	Glucose-responsive transcription factor
YDL138W	*RGT2*	plasma membrane	Plasma membrane glucose sensor
YDL194W	*SNF3*	plasma membrane	Plasma membrane low glucose sensor
YHR094C	*HXT1*	plasma membrane	Low-affinity glucose transporter
YMR011W	*HXT2*	plasma membrane	High-affinity glucose transporter
YHR092C	*HXT4*	plasma membrane	High-affinity glucose transporter

The gene expression of *RGT1*, *RGT2*, and *SNF3*, which encode a component of a transcriptional regulator complex and glucose sensors, showed similar patterns to other transcriptional regulators such as *PDR1* and *PDR3*. The inhibitory regulation of *RGT1* on *HXT* genes is abolished by glucose sensing. However, the cells were cultured in YPD with each mycotoxin for only 2 h, so there was a low possibility for glucose deficiency in the culture media. Because no glucose was supplied in the test conditions, the glucose transporter genes might have been regulated by a different trigger to glucose. Differences in gene expression trends between the three mycotoxins that were not linked to these regulatory genes directly suggested abnormal regulation mechanisms. Essentially, 15ADON exposure leads to higher toxicity than that of DON in yeast cell studies, and both DON and 15ADON induce similar trends of gene expression changes. However, for the *HXT* genes in this research, 15ADON showed negative correlations and DON induced these genes under all conditions, which was unusual. Taken together, this suggests that DON and 15ADON do not have completely corresponding toxicity.

## 3. Conclusions

As of 2010, the provisional maximum tolerable daily intake (PMTDI) established for DON was extended to acetyl-DON by the Joint FAO/WHO Expert Committee on Food Additives (JECFA) [32]. This is because acetyl-DON is metabolized in the body and the de-acetylation product is synthesized such that acetyl-DON is thought to potentially have the same risk as DON. However, the intestinal surface around the duodenum is at risk of being damaged by acetylated products directly because the de-acetylation reaction has not progressed or started yet. Therefore, understanding the differences in toxicity between DON and its acetylated products is important for the development of toxicity evaluation. This study suggests that the toxic effects of DON partially differ from those of 15ADON; thus, further investigation of the toxicity of such modified products is needed.

## 4. Experimental Section

### 4.1. Chemicals

Deoxynivalenol (DON), 3-acetyldeoxynivalenol (3ADON), and 15-acetyldeoxynivalenol (15ADON) powders (Wako, Osaka, Japan) were each dissolved in dimethyl sulfoxide (DMSO). Stock solutions were added into each medium to prepare test conditions.

### 4.2. Cell Strain and Growth Conditions

A *Saccharomyces cerevisiae* BY4743 (*MATa/α his3Δ1/his3Δ1 leu2Δ0/leu2Δ0 LYS2/lys2Δ0 met15Δ0/MET15* *ura3Δ0/ura3Δ0*) derived *PDR5* deletion mutant (*pdr5Δ*; Thermo Fisher Scientific, Waltham, MA, USA) with the loss of multidrug resistance transporter function was used in this study. Glycerol stocks, which were stored at −80 °C, of yeast cells were transferred with an inoculating needle into YPD media (1% yeast extract, 2% peptone, and 2% glucose) into three conical flasks and incubated on a rotary shaker (TOKYO RIKAKIKAI, Tokyo, Japan) at 150 rpm and 25 °C for at least 2 days. The pre-culture solutions were measured with a spectrophotometer at 650 nm. These solutions were transferred to fresh YPD to dilute the cell concentration, and were incubated on a rotary shaker at 150 rpm and 25 °C for several hours until the absorbance at 650 nm (A_650_) = 0.8–1.0. Six milliliter aliquots of the prepared culture solutions were dispensed into test tubes, and a final concentration of 0.01% sodium dodecyl sulfate was added to enhance the cell permeability of the mycotoxins. The mycotoxins were added into the test tubes at final concentrations of 5, 10, 25, 50, and 100 mg/L. DMSO, which was used to dissolve the mycotoxins, was added to a non-treatment control sample (0 mg/L). DMSO was also added to other samples so that the final concentration of each additive was 0.5%. The prepared test tube samples were immediately incubated on a reciprocal shaker (TAITEC, Saitama, Japan) at 150 strokes/min and 25 °C for 2 h. The culture media were transferred into 15 mL corning tubes, and cell pellets were obtained by centrifugation at 3000 rpm for 7 min at 4 °C. The pellets were immediately frozen with liquid nitrogen, and stored at −80 °C.

### 4.3. RNA and cDNA Preparation, Semi-Quantitative PCR

Total RNA was extracted from the cell pellets using a commercial kit (FastRNA Pro Red kit, MP Biomedicals, Irvine, CA, USA), following the manufacturer’s instructions. To avoid genomic DNA contamination, the samples were treated with deoxyribonuclease (DNase I, Takara, Shiga, Japan) before reverse transcription. First-strand cloned DNA (cDNA) was synthesized with reverse transcriptase (Prime script II 1st strand cDNA synthesis kit, Takara, Japan) from the total RNA. Residual RNAs in the cDNA products were digested with ribonuclease A (RNaseA, Thermo Fisher Scientific, Waltham, MA, USA). Each cDNA product was diluted with two volumes of Tris-EDTA (pH 8.0), and the cDNA solution was used as a template. Sequence information for the target genes was obtained from the Saccharomyces Genome Database (SGD) [33], and each primer set that corresponded to the position around the 3′-end sequence was manually designed (Table 5). The actin protein coding gene *ACT1* was used as an internal control. An *ACT1* primer set with the same sequence as in a previous report [34] was prepared. The other primer sets were designed by Primer3 [35]. To obtain a standard curve for each target gene, non-treatment control (NTC) cDNA was prepared and PCR was conducted to construct the standard curves. In this study, 20 μL reaction solutions (1 μL of each 10 μM primer, 2 μL of 10× buffer, 1.6 μL of 2.5 mM dNTPs, 1 μL of DNA template, 15.2 μL of distilled water, and 0.2 μL of Taq polymerase (Gene Taq, Nippon Gene, Tokyo, Japan) were prepared and subjected to 95 °C for 5 min, followed by 35 cycles of 95 °C for 30 s, 55 °C for 30 s, and 72 °C for 60 s in a thermal cycler (C1000, Bio-Rad, Hercules, CA, USA). For each primer set, a template dilution series was prepared from the PCR products and 1 μL of each sample was dispensed into a 96-well PCR plate. The same volume of mycotoxin-treated sample templates were also dispensed into the plate. A total of 19 μL of reaction mix (0.4 μL of each 10 μM primer, 8.2 μL of distilled water, and 10 μL of 2× Master mix (KAPA SYBR FAST qPCR kit, Kapa Biosystems, MA, USA)) was added to each well. The sample plate was subjected to 95 °C for 20 s, followed by 40 cycles of 95 °C for 3 s, and 60 °C for 20 s, in a thermal cycler (MX3000P, Agilent Technologies, Santa Clara, CA, USA). The amplified *ACT1* product was used as an internal control, and triplicates were averaged. Part of a DNA microarray dataset (accession no. GSE36954) recorded in the Gene Expression Omnibus Database (GEO) was also used [36]. Two-way factorial ANOVA was conducted for each gene expression data series, and, under some concentration conditions, two facultative mycotoxin-exposed samples were compared by Student’s *t*-test analysis.

**Table 5:** Primer sets used in this study.

Name	Direction	Forward or Reverse
	5′ 3′	
*ACT1*	ATTGCCGAAAGAATGCAAAAGG	F
	CGCACAAAAGCAGAGATTAGAAACA	R
*FRE1*	GCTCGGAAATAAAACTCTCAGAA	F
	ATTATTAACAAGGGGCCTTACCA	R
*FRE2*	CGACCAAATGTTAAGGAACTTCTAC	F
	AAATGATCACCAGCATTGATACTCT	R
*FRE3*	TAAATGGATCGTTAGCTGTGGTT	F
	TTGTGATAGGTAAAATAGTGAGGAAA	R
*FRE7*	GCTTCATTTGTTCAGGTTCTGAC	F
	TGCTTGAATGATATTTCACATGG	R
*FRE8*	ACGGATTCTATTCAATGTTGCAG	F
	TCAAATACGTGAATTTTCCAAGC	R
*FUS3*	ATCCGTATTTGCAAACATACCAC	F
	TGTATACATTGTTCTTCGGGTTGATA	R
*HXT1*	CGTCTTTTTCTTCGTTCCAGA	F
	GAGCTTGTTTAGTTTATTTCCTGCT	R
*HXT2*	GGGTATGTCTTCATGGGCTGT	F
	TATAATCTCTTATTCCTCGGAAACTC	R
*HXT4*	TTCTTCGTTCCAGAAACTAAAGG	F
	CCGAACATCTTCTTGTAAAATGG	R
*INO1*	AAGATGCTGGCAAATTCGAG	F
	TGAGATTACAACAATCTCTCTTCG	R
*PDR1*	ACAATATTAACAACAACAACAGTAACAA	F
	GGAAGGAAGTTTTTGAGAACTTTTA	R
*PDR3*	CAACAGACAAAAAGACAACATTCTG	F
	CCATTTACTATGGTTATGCTCTGCT	R
*RGT1*	CCTCCGCGAGTCATCAGT	F
	ACCTGTCAATACCAGCCTAACTC	R
*RGT2*	CAATAACAACACTGAACGAAATGG	F
	GGGGAAGTGTATTGGCTGTG	R
*SNF3*	GAACGAATGGCGCAGTTT	F
	CAAATCATTATTTTCATTTACAGGTTG	R
*SNQ2*	GCTACTTGTGGAGAAATTTTGGA	F
	GCAGATGAATGCACAAAATGTTA	R
YLR307C-A	GGGATGCTAGTACTACTCAATGTCG	F
	TTAATTCAAATTATACTTTTACGTGCTC	R
YMR230W-A	GGATGTGTTACGATGCAGACA	F
	ATATGGCGCGTTCTTGAAGG	R

## Nomenclature

Three capital letters with *Italics*; gene

Two lowercase letters following a capital letter + p; coding protein

Three small letters with *Italics* + *Δ*; mutant strain

ANOVA; analysis of variance
